# The Exometabolome of Two Model Strains of the *Roseobacter* Group: A Marketplace of Microbial Metabolites

**DOI:** 10.3389/fmicb.2017.01985

**Published:** 2017-10-12

**Authors:** Gerrit Wienhausen, Beatriz E. Noriega-Ortega, Jutta Niggemann, Thorsten Dittmar, Meinhard Simon

**Affiliations:** Institute for Chemistry and Biology of the Marine Environment, University of Oldenburg, Oldenburg, Germany

**Keywords:** roseobacter, DOM, exometabolome, black queen hypothesis

## Abstract

Recent studies applying Fourier transform ion cyclotron resonance mass spectrometry (FT-ICR-MS) showed that the exometabolome of marine bacteria is composed of a surprisingly high molecular diversity. To shed more light on how this diversity is generated we examined the exometabolome of two model strains of the *Roseobacter* group, *Phaeobacter inhibens* and *Dinoroseobacter shibae*, grown on glutamate, glucose, acetate or succinate by FT-ICR-MS. We detected 2,767 and 3,354 molecular formulas in the exometabolome of each strain and 67 and 84 matched genome-predicted metabolites of *P. inhibens* and *D. shibae*, respectively. The annotated compounds include late precursors of biosynthetic pathways of vitamins B_1_, B_2_, B_5_, B_6_, B_7_, B_12_, amino acids, quorum sensing-related compounds, indole acetic acid and methyl-(indole-3-yl) acetic acid. Several formulas were also found in phytoplankton blooms. To shed more light on the effects of some of the precursors we supplemented two B_1_ prototrophic diatoms with the detected precursor of vitamin B_1_ HET (4-methyl-5-(β-hydroxyethyl)thiazole) and HMP (4-amino-5-hydroxymethyl-2-methylpyrimidine) and found that their growth was stimulated. Our findings indicate that both strains and other bacteria excreting a similar wealth of metabolites may function as important helpers to auxotrophic and prototrophic marine microbes by supplying growth factors and biosynthetic precursors.

## Introduction

The biochemical processes in a living and active prokaryotic cell yield a highly complex blend of metabolites reflecting the catabolic, metabolic and anabolic properties of the organism. On the basis of available genomic information on the metabolic potential, metabolite patterns within the cell, endometabolomics, have been investigated during the recent past in various prokaryotes and as a function of substrate source and growth conditions (Rosselló-Mora et al., 2008; Zech et al., 2009; Frimmersdorf et al., 2010; Paczia et al., 2012; Drüppel et al., 2014). The exometabolome, i.e. the pool of metabolites released into the cell's environment, has also been investigated, showing species- or even ecotype-specific fingerprints as a function of growth stage and conditions (Kell et al., 2005; Villas-Bôas et al., 2006; Paul et al., 2009; Paczia et al., 2012; Romano et al., 2014; Fiore et al., 2015; Johnson et al., 2016).

Most of these studies applied targeted approaches mainly gas chromatography–mass spectrometry (GC–MS), searching for metabolites to be expected from predicted substrate use and metabolic pathways (Villas-Bôas et al., 2006; Zech et al., 2009; Drüppel et al., 2014). Several studies have used Fourier transform ion cyclotron resonance mass spectrometry (FT-ICR-MS) applying non-targeted approaches and some combined both. They found that the diversity of the endo- and in particular the exometabolome is far higher than expected from targeted approaches, yielding several thousand molecular masses (Rosselló-Mora et al., 2008; Brito-Echeverría et al., 2011; Romano et al., 2014; Fiore et al., 2015; Johnson et al., 2016). Besides the expected metabolites, the metabolome obviously includes a surprisingly high proportion of compounds not expected from predicted metabolic pathways. In particular the exometabolome exhibited a wealth of metabolites, many with so far unknown molecular masses and elemental composition (Rosselló-Mora et al., 2008; Kujawinski et al., 2009; Romano et al., 2014; Fiore et al., 2015). There is some evidence that quite a few of these metabolites are released as a result of an overflow metabolism due to growth on an abundant carbon source (Paczia et al., 2012; Romano et al., 2014). However, under less excessive growth conditions, in addition to well-known exometabolites such as signaling compounds (Dickschat, 2010; Hartmann and Schikora, 2012), vitamins (Sañudo-Wilhelmy et al., 2014), siderophores (Mansson et al., 2011), many other unexpected metabolites are released into the environment. The bacterial secretion of several such microbial metabolites, including the plant hormone indole 3-acetic acid (IAA) and vitamin precursors, has been documented (Zhang et al., 2014; Amin et al., 2015; Fiore et al., 2015; Johnson et al., 2016; Paerl et al., 2017). Analyses of dissolved organic matter (DOM) from Sargasso Sea waters detected several compounds with molecular masses known to be released by a strain of the globally abundant SAR11 clade (Kujawinski et al., 2009). The thiamine (vitamin B_1_) precursor 4-amino-5-hydroxymethyl-2-methylpyrimidine (HMP) was also detected. This precursor is essential for thiamine biosynthesis by members of the SAR11 clade because they lack the gene for its complete biosynthesis (Carini et al., 2014). Thiamine requirements by photoautotrophic eukaryotes can also be met when the phosphorylated form, thiamine diphosphate, is dephosphorylated by a partner. In a bioassay approach Paerl et al. (2015) showed that the thiamine-auxotrophic picoeukaryote *Ostreococcus* sp. could grow when supplied with thiamine diphosphate in the presence of an *Alteromonas* strain that exhibit phosphatase activity.

The exchange of metabolites and precursors between microbes appears to be more common because auxotrophy is much wider distributed among microbes than previously assumed (McRose et al., 2014; Garcia et al., 2015; Paerl et al., 2017). Growth of such organisms depends on mutualistic interactions and supply of metabolites by co-occurring microbes. The Black Queen hypothesis applied this phenomenon to explain genome streamlining of prokaryotes by deleting certain metabolic pathways or parts of it as a reaction to leaky metabolic pathways of other microbes, helpers, thus supplying metabolites or precursors as public goods to the auxotrophic prokaryotes (Morris et al., 2012). It has been proposed most recently that gene loss and niche partitioning may be major drivers in the co-evolution of auxotrophs and helpers (Mas et al., 2016). However, considering the metabolites found in the exometabolome of various microbes and the environment in recent studies it appears that exchange of metabolites has larger ramifications than just explaining genome streamlining of selected microbes (Zelezniak et al., 2015; Estrela et al., 2016). We hypothesize that the exometabolome of helpers includes multiple metabolites and precursors, not only vitamins and growth factors, which are beneficial for other microbes. This release may promote growth of auxotrophic organism and even enhance growth of prototrophic microbes because they may not need to allocate energy to synthesize these precursors.

In order to test this hypothesis we assessed the exometabolome of two model bacteria of the marine *Roseobacter* group, *Phaeobacter inhibens* DSM 17395 and *Dinoroseobacter shibae* DSM 16493, growing on either glucose, glutamate and acetate or succinate, by analysis on a 15 Tesla FT-ICR-MS. *Phaeobacter inhibens* DSM 17395 is a purely heterotrophic bacterium growing in biofilms (Thole et al., 2012; Gram et al., 2015). Strains of this species and the genus *Phaeobacter* have been found in various marine habitats, such as natural biofilms on solid surfaces and during a bloom of *Emiliana huxleyi* (Gifford et al., 2014; Gram et al., 2015; Segev et al., 2016; Breider et al., 2017). *Dinoroseobacter shibae* is photoheterotrophic, grows symbiotically with dinoflagellates (Wagner-Döbler et al., 2010), produces various signaling compounds (Neumann et al., 2013) and has also been found associated to algae in natural phytoplankton blooms (Gifford et al., 2014; Milici et al., 2016; Segev et al., 2016). In order to examine whether identified molecular masses, and more precisely formulas, are also relevant under natural conditions, we screened DOM samples analyzed by FT-ICR-MS from a mesocosm experiment (Osterholz et al., 2015) and the North Sea. It is the first application of such a powerful FT-ICR-MS for exometabolomic studies in combination with the search for molecular formulas in environmental samples thus greatly enhancing the sensitivity and resolution of metabolite identification.

## Materials and methods

### Growth conditions

*Phaeobacter inhibens* DSM 17395 and *D. shibae* DSM 16493 were first grown on marine broth (MB; Difco MB 2216) medium and afterwards repeatedly (5x) transferred and cultivated in artificial seawater (ASW) medium with the addition of a single organic carbon source. Before every transfer and after centrifugation of the cultures the cell pellets were washed three times with ASW medium. All plastic and glass ware used were rinsed with acidified ultrapure water (MilliQ, pH 2) and all glassware additionally combusted for 3 h at 500°C.

The ASW-medium for the *P. inhibens* cultures was prepared as described by Zech et al. (2009) but slightly modified by excluding EDTA from the trace element solution. The cultures were supplemented with glucose (5 mM, 30 mM C; ultrapure brand), acetate (30 mM, 60 mM C; ultrapure brand) or glutamate (20 mM, 100 mM C; ultrapure brand). *Dinoroseobacter shibae* was cultivated in ASW-medium (Soora and Cypionka, 2013) with the same concentrations of glucose as for *P. inhibens* and of glutamate at 7.5 mM (37.5 mM C). Instead of acetate, succinate was used at an initial concentration of 10 mM (40 mM C; ultrapure brand). Concentrations of the substrates were adjusted due to varying metabolic rate efficiencies to obtain rather equal growth yields as determined by optical density (OD_600_). Bacteria were cultivated in 500 ml of medium in triplicate 2 liter baffled Erlenmeyer glass flasks at pH 8 at 28°C in the dark on a shaker (100 rpm) and growth was monitored by OD. A sterile flask with media and the respective single carbon source was run as control. Subsamples were withdrawn under laminar flow for the separate analysis of the replicates for dissolved organic carbon, exometabolome-DOM, dissolved free (DFAA) and dissolved combined amino acids (DCAA), dissolved free neutral monosaccharides (DFNCHO), and dissolved combined monosaccharides (DCNCHO) and fatty acids at the start, the lag phase, the mid-exponential and early stationary phase in order to recover the majority of exometabolites released at varying growth conditions. The growth patterns were assessed more precisely than by OD measurements by subsampling every 4–8 h depending on the growth phase patterns for bacterial cell enumeration. Cells were fixed with 2% glutaraldehyde and frozen at −20°C until further analysis.

In order to test the effect of the vitamin B_1_ precursors HMP (AstaTech inc., Bristol, PA, USA) and HET [4-methyl-5-(β-hydroxyethyl)thiazole; Sigma Aldrich, Munich, Germany] on the growth of the diatoms *Thalassiosira pseudonana* (CCMP 1335) and *Leptocylindrus danicus* (CCMP 470) these diatoms were grown axenically in ASW medium in a 12:12 h light dark cycle and illuminated at 70 μE. Instead of B_1_, the precursors were added at 100 nM final concentration together with vitamins B_7_ and B_12_ (100 nM each) and growth was monitored as relative fluorescence against a positive control including all three vitamins at final concentrations of 100 nM each and a negative control including only B_7_ and B_12_ at the same concentrations. Axenicity of the diatom cultures was checked microscopically.

### Cell abundance

Cells of the *D. shibae* cultures were enumerated by flow cytometry, those of the *P. inhibens* cultures by epifluorescence microscopy due to the formation of microaggregates. Flow cytometric analyses were done according to Osterholz et al. (2015). Cell aggregates of *P. inhibens* were dispersed by ultrasonication (5 × 10 s at 15 mV; Bandelin Sonopuls HD 200, Bandelin, Berlin, Germany), filtered onto a 0.2 μm polycarbonate membrane, stained for 30 min with SYBR® Green I and counted by epifluorescence microscopy as described (Lunau et al., 2005). At least 1,000 cells were enumerated per filter.

### Amino acids, mono- and polysaccharides and fatty acids

Aliquots of the cultures were centrifuged at 2,499 g in acid washed and combusted (3 h, 500°C) glass centrifuge tubes. The supernatant was filtered through a 0.22 μm polyethersulfone membrane (Minisart, Sartorius, Göttingen, Germany) and the filtrate stored in combusted 20 ml glass vials at −20°C until further analysis. Concentrations of DFAA and DCAA were analyzed by high performance liquid chromatography (HPLC) after precolumn derivatization with orthophtaldialdehyde (Lunau et al., 2006) and concentrations of DFNCHO and DCNCHO by HPLC and pulsed amperometric detection after desalting (Hahnke et al., 2013). Detection limits for DFAA and DFNCHO were 0.5 and 1.5 nM, respectively. Fatty acid concentrations in the treatments with additions of succinate and acetate were determined by HPLC (Sykam, Fürstenfeldbruck, Germany) equipped with an Aminex HPX-87H column (Biorad, München, Germany) (Graue et al., 2012).

### DOM analyses

Dissolved organic carbon in the filtrates of the bacterial cultures and of the solid-phase extracted DOM (see below) was quantified as described previously (Osterholz et al., 2014). For FT-ICR-MS analyses, filtrates were acidified to pH 2 (HCl 25% p.a., Carl Roth, Germany), extracted via PPL solid phase cartridges (100 mg; Agilent, Waldbronn, Germany) adapted to a concentration of 15 ppm carbon and analyzed by FT-ICR-MS according to Osterholz et al. (2015). The extraction efficiency increased from 2 to 31% on carbon basis in the course of the experiment, as result of the contrary running substrate availability. Extracted DOM was ionized by soft electrospray ionization (Bruker Apollo, Daltonics, Bremen, Germany) and analyzed in positive and negative mode with a 15 T Solarix FT-ICR-MS (Bruker, Daltonics, Bremen, Germany). For each spectrum 300 scans were accumulated in the mass window of 92 to 2,000 Da. An internal calibration list was generated using Bruker Daltonic Data Analysis software for the calibration of the spectra. FT-ICR-MS instrument performance was verified using a laboratory-internal deep ocean DOM reference sample. Detected mass to charge ratios were processed applying a customized routine Matlab script. Molecular formulas were assigned as described by Koch and Dittmar (2006) to molecular masses with a minimum signal-to-noise ratio of 5 (Koch et al., 2007). From the mass spectrograms of the single time points of each culture that of the respective sterile control was subtracted.

### Exometabolite fragmentation

To confirm structures of genome-predicted identified exometabolites, we performed fragmentation experiments using FT-ICR-MS. Therefore, both bacterial strains were cultured again at similar conditions as described above for the exometabolome experiments but the exometabolome was harvested at the time point of the peak concentration of the respective exometabolite. A total of 500 mL was extracted via PPL solid phase cartridges as described above. Extracts were redissolved in a 1:1 MilliQ water/methanol solution at a concentration of 29 ppm carbon and analyzed on the FT-ICR-MS as described above. We selected the exometabolites which were identified as precursors or products of biosynthetic pathways for fragmentation, i.e., 43 of the 107 exometabolites identified in total. However, only seven metabolites had sufficiently high signal intensities in the FT-ICR-MS to perform fragmentation experiments. Limitations to fragmentation experiments include the fact that fragments may not be ionizable and different fragments with rather similar masses may yield overlapping peaks. Exometabolites of interest were isolated in a 1 Da window using the quadrupole unit and collision with argon occurred in the hexapole unit of the FT-ICR-MS. Fragmentation parameters were optimized for each mass with an isolation window ranging from 0.1 to 1 (m/z), collision energy adjusted by applying 10 to 15 mV and 300 to 700 broadband scans were accumulated per run.

### Exometabolite prediction from genome databases and screening against natural DOM samples

Metabolites of *P. inhibens* and *D. shibae*, predicted by the genome database BioCyc (Caspi et al., 2012), were listed with their corresponding molecular masses and molecular formulae (MF). All MF calculated from FT-ICR-MS detected masses were scanned against the genome-based metabolite prediction list. Matches were analyzed in more detail, regarding intra and extracellular function identified by previous studies. Putatively identified MF of exometabolites were screened against MF of DOM data sets of naturally derived and North Sea phytoplankton blooms (Osterholz et al., 2015, 2016; Noriega-Ortega et al., in preparation).

## Results

Both model strains of the *Roseobacter* group were grown in batch culture on single carbon sources of three major substrate classes to examine the diversification of the exometabolome as a function of these different substrates. Here we focus on the identified exometabolites whereas the overall exometabolome and its diversity is dealt with in a different publication (Noriega-Ortega et al., in preparation)

### Growth and substrate utilization

**Figure 1:**
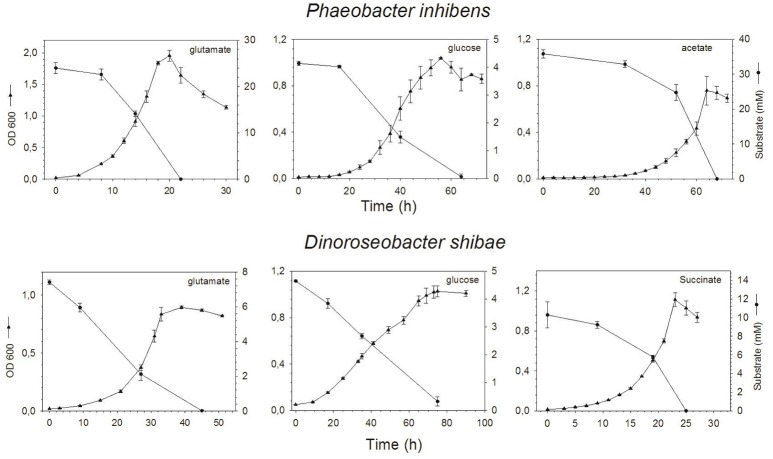
Optical density and concentration of the substrates glutamate, glucose and acetate or succinate over time of *P. inhibens* **(upper)** and *D. shibae* **(lower)**. Note the different scales of the axes.

*Phaeobacter inhibens* reached highest growth with maximum ODs of 0.76, 1.04, and 1.96 on acetate, glucose and glutamate respectively, at 68, 56, and 20 h (Figure 1). Respective cell numbers at these time points were 1.4 × 10^9^, 0.6 × 10^9^, and 3.4 × 10^9^ cells mL^−1^ (see supporting information Figure S1). Growth of *D. shibae* reached highest ODs of 1.11, 1.03, and 0.90 on succinate, glucose and glutamate respectively, at 23, 65, and 39 h (Figure 1) with corresponding cell numbers of 2.1 × 10^9^, 0.8 × 10^9^, and 3.3 × 10^9^ cells mL^−1^ respectively (see supporting information Figure S1). Substrate concentrations decreased inversely to growth of both bacteria and went below detection limit in the stationary phase except for the culture of *D. shibae* growing on glucose (Figure 1).

### Amino acids in the exometabolome

**Figure 2:**
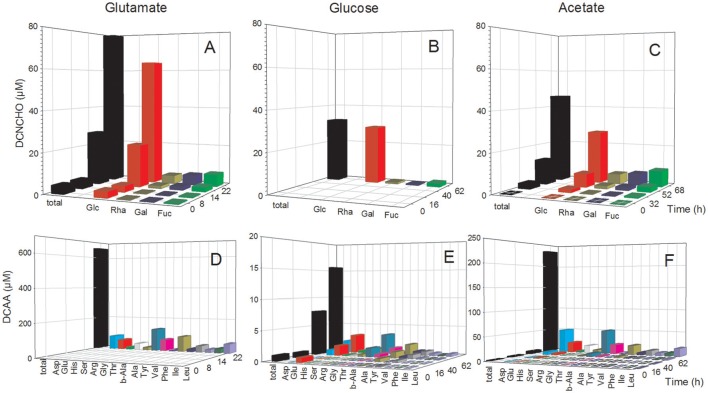
Concentrations of individual neutral monosaccharides and amino acids (AA) bound in total dissolved combined neutral monosaccharides (DCNCHO) and total dissolved combined amino acids (DCAA), respectively, in the *P. inhibens* cultures. DCNCHO **(A–C)** and DCAA **(D–F)** right after inoculation, in the lag, exponential and stationary phase of *P. inhibens* growing on glutamate **(A,D)**, glucose **(B,E)** and acetate **(C,F)**. Note the different scales of the parameters in the different panels. Concentrations of DCNCHO in the treatment with glucose are not available until the stationary phase at 62 h due to interference with the HPLC analysis. Concentrations of AA in the glutamate treatment are only available for 22 h in the stationary phase because high glutamate concentrations until the exponential phase interfered with the HPLC analysis.

The analysis of dissolved amino acids within the exometabolome of both strains was biased in the glutamate treatments by the interfering large glutamate peak. Hence, concentrations could only be measured in the stationary phase when glutamate was completely utilized. In the *P. inhibens* cultures, concentrations of DFAA remained below the detection limit of the HPLC analysis at all growth phases but tryptophan, tyrosine and histidine were detected by the FT-ICR-MS analysis (see below). Concentrations of DCAA in the treatment with acetate remained below 4 μM during the exponential growth phase but reached 235.4 μM in the stationary phase (Figure 2F). In the treatment with glucose, DCAA concentrations increased continuously during the exponential and stationary phase but reached a final concentration of only 15.6 μM (Figure 2E). The glutamate treatment of this culture yielded a concentration of 684.9 μM in the stationary phase (Figure 2D). The substrate source had only a minor influence on the composition of the amino acid pool in the exometabolome. During the exponential and stationary phases aspartate, glutamate, glycine, and alanine constituted the highest mol% of DCAA and together at least 60 mol% (Figure 2, Table S1). The very high DCAA concentrations in the exometabolome may have been a result of cell lysis. To estimate the extent of potential lysis, we made a mass balance of carbon (C) in DCAA and in the bacterial biomass in the stationary phase. DCAA concentrations of 219.8 and 639.0 μM in the treatments with acetate and glutamate in the stationary phase translate into 11.5 and 32.8 mg C L^−1^, respectively. On the basis of 50 fg C per cell of large bacteria (Simon and Azam, 1989), typical for fast growing cultures, the bacterial numbers of 1.39 × 10^9^ and 3.44 × 10^9^ cells mL^−1^ in the stationary phase in the acetate and glutamate treatments, respectively, equal 69.5 and 160.9 mg C L^−1^. Hence, the C bound in DCAA comprises 17.1 and 20.6% of the C bound in bacterial biomass. Therefore, we conclude that at these two conditions, but neither at other conditions nor in the glucose treatments, protein released by lysed cells contributed to the high DCAA concentrations in the stationary phase of the *P. inhibens* culture. Consequently, these time points were not considered for further exometabolome analyses.

**Figure 3:**
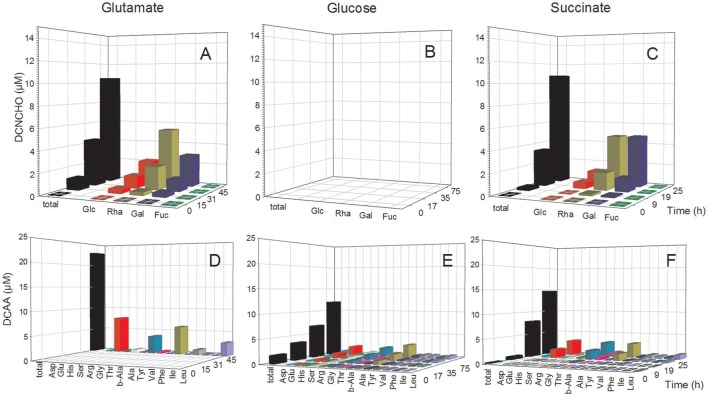
Concentrations of individual neutral monosaccharides and amino acids (AA) bound in total dissolved combined neutral monosaccharides (DCNCHO) and total dissolved combined amino acids (DCAA), respectively, in the *D. shibae* cultures. DCNCHO **(A–C)** and DCAA **(D–F)** right after inoculation, in the lag, exponential and stationary phase of *D. shibae* growing on glutamate **(A,D)**, glucose **(B,E)** and succinate **(C,F)**. Concentrations of CHO in the treatment with glucose are not available due to interference with the HPLC analysis. Concentrations of DCAA in the glutamate treatment are only available for 45 h in the stationary phase because high glutamate concentrations until 31 h interfered with the HPLC analysis.

In the *D. shibae* cultures, DFAA concentrations were below the detection limit of the HPLC analyses at all growth phases, but tryptophan, tyrosine, phenylalanine and histidine were detected by FT-ICR-MS (see below). Concentrations of DCAA in the exometabolome of the *D. shibae* cultures continuously increased during the exponential growth phase and reached 12.0 and 14.7 μM in the glucose and succinate treatments in the stationary phase (Figure 3). The glutamate treatment yielded 23.0 μM in the stationary phase (Figure 3D). Glutamate, glycine and alanine dominated the DCAA pool at all substrate conditions and constituted >50 mol% (Table S2). A mass balance of carbon bound in DCAA and the bacterial biomass in the stationary phase of the three treatments showed that DCAA constituted always <1% of the C bound in the biomass of the *D. shibae* cultures. Hence, cell lysis was minimal in these cultures.

### Mono- and polysaccharides in the exometabolome

The analysis of dissolved mono- and polysaccharides interfered with the addition of glucose as single substrate source. DFNCHO were not detected in the exometabolome at any growth stage of both strains. Concentrations of DCNCHO increased continuously during growth of both strains on glutamate and acetate or succinate with highest concentrations in the stationary phase (Figures 2, 3). Released DCNCHO in the *P. inhibens* cultures were greatly dominated by glucose but galactose, rhamnose, and fucose constituted proportions of up to 22 mol% in the treatment with acetate (Figure 2, Table S2). In the *D. shibae* cultures, DCNCHO concentrations remained lower than in those of *P. inhibens* (Figure 3). Only glucose, galactose and rhamnose were detected in the exometabolome. In the succinate treatment, rhamnose and galactose dominated whereas in the glutamate treatment galactose became the dominant DCNCHO component in the exponential and stationary phase (Table S2).

### Exometabolome diversity and exometabolite identification

**Figure 4:**
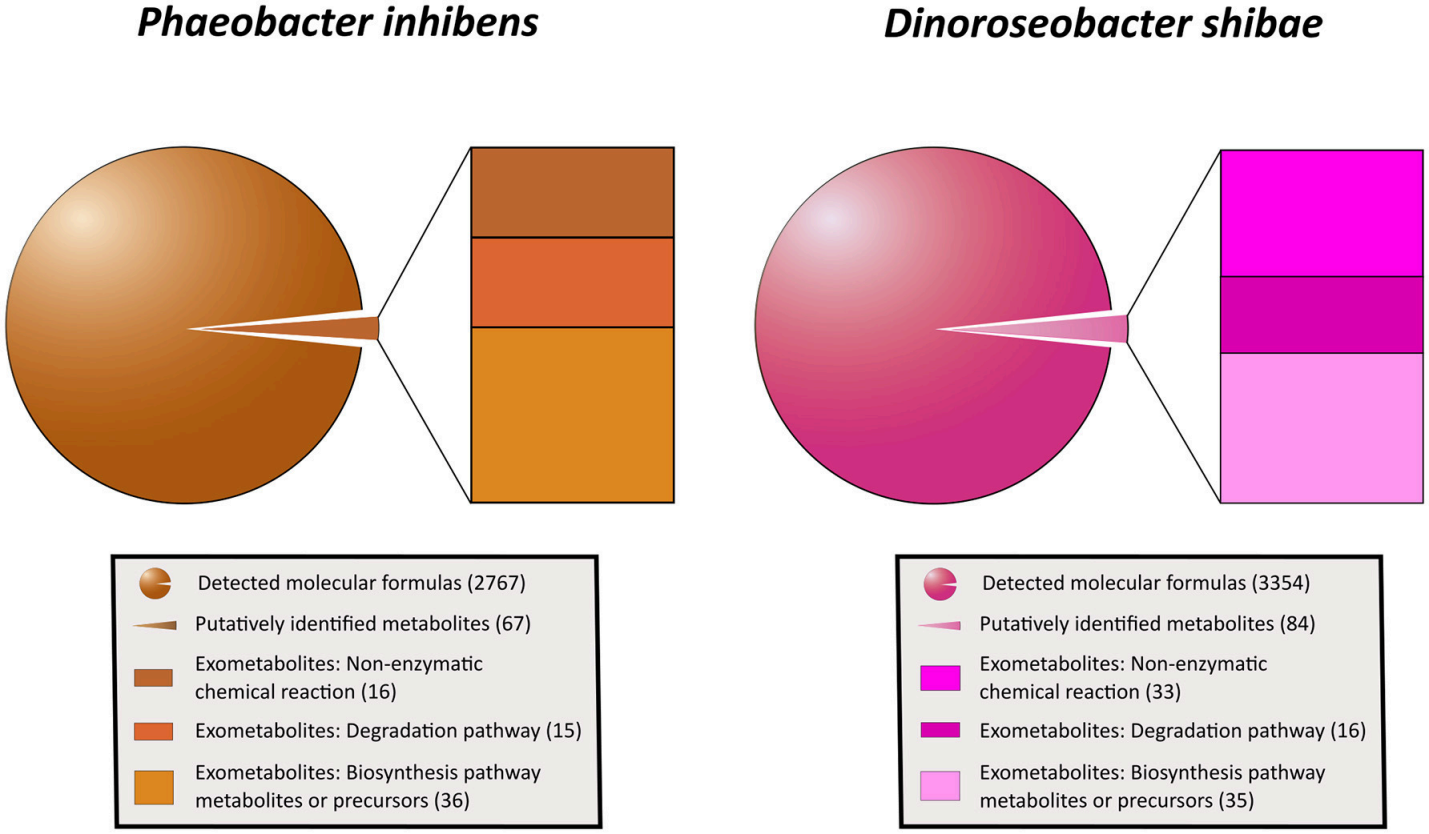
Subdivision of all exometabolites released by *P. inhibens* and *D. shibae* during all growth phases. Given (in parenthesis) are total numbers of detected molecular formulas, putatively identified metabolites and those related to non-enzymatic chemical reactions, degradation and biosynthetic pathways and precursors.

Applying the ultrahigh resolution FT-ICR-MS, we detected in total 2767 MF in the exometabolome of *P. inhibens* and 3,354 MF in that of *D. shibae* (Figure 4; Tables S3, S4). These numbers include all growth stages and all substrate treatments of both strains, except the stationary phase of *P. inhibens* grown on glutamate and acetate due to suspected cell lysis. The composition of the exometabolome, but not the exometabolites identified (see below) of both strains varied considerably as a function of the substrate utilized and growth stage (Noriega-Ortega et al., in preparation). Scanning all detected exometabolomic MF against the genome-predicted metabolites of *P. inhibens* and *D. shibae* by applying the BioCyc database revealed a match for 67 and 84 exometabolites, respectively (Figure 4). In addition, secondary metabolites known to be produced by *P. inhibens* and *D. shibae* were scanned against the exometabolomic MF obtained by FT-ICR-MS, yielding 4 identified compounds in the exometabolome of *P. inhibens* but none in that of *D. shibae* (Table 1). Identified exometabolites were further subdivided on the basis of their function and occurrence within the bacterial metabolism (Figure 4). For *D. shibae*, 35 metabolites were assigned to biosynthetic pathways (Table 1), 16 metabolites to degradation pathways (Table S5) and 33 metabolites originated from spontaneous non-enzymatic chemical-reactions (Table S6). For *P. inhibens*, 36 metabolites were assigned to biosynthetic pathways (Table 1), 15 to degradation pathways (Table S5), but only 16 derived from spontaneous non-enzymatic chemical reactions (Table S6). In total 43 different exometabolites of biosynthetic pathways were identified of which 28 were present in the exometabolome of both strains. In the *D. shibae* experiments 19 exametabolites assigned to biosynthetic pathways were detected in >50% of the time points sampled and in the experiments with *P. inhibens* 16 exometabolites. Alpha-ribazol was detected only at three time points in the exometabolome of *D. shibae* but in 88% of the time points of the *P. inhibens* experiments. Pyridoxal-P and histidine were detected only once in the exometabolome of the *D. shibae* experiments but in 43 and 55% of that of the *P. inhibens* experiments, respectively. HET was detected only once in the exometabolome of *P. inhibens* experiments but in 58% of that of the *D. shibae* experiments. All other exometabolites were detected in 10–50% of the samples analyzed in the experiments of both strains.

**Table 1:** Metabolites, their molecular formula, function, fragmentation results detected in the exometabolome of *D. shibae* and *P. inhibens*, in other studies, a mesocosm (Osterholz et al., 2015) and in a North Sea phytoplankton bloom (Noriega-Ortega et al., in preparation).

**Metabolites**	**Molecular formula**	**Function**	**Fragmentation**	***D. shibae***	***P. inhibens***	**Other studies**	**Mesocosm**	**North Sea bloom**
HET (4-methyl-5-(β-hydroxyethyl)thiazole)	C6H9NOS	vitamin B1 precursor		+	+			
Thiamine phosphate	C10H15N2O8P	vitamin B1		+	+	G, J		
Dimethyl-D-ribityl-lumazine	C13H18N4O6	vitamin B2 precursor	+	+	+	R		
Riboflavin	C17H20N4O6	vitamin B2	+	+	+	F, R, G, J		
Pantoate	C6H12O4	vitamin B5 precursor		+				
Pantothenate	C9H17NO5	vitamin B5		+	+	G, J		
Pyridoxal	C8H9NO3	vitamin B6 related		+	+		+	+
Pyridoxal phosphate	C8H10NO6P	vitamin B6		+	+			
Dethiobiotin	C10H17N2O3	vitamin B7 precursor			+			
Alpha-ribazole	C14H18N2O4	vitamin B12 precursor	+	+	+	R, J	+	+
Alpha-ribazole-5-phosphate	C14H19N2O7P	vitamin B12 precursor		+	+			+
Pyrroloquinoline quinone	C14H6N2O8	vit. B - cofactor	+	+				
6-(2-amino-2-carboxylatoethyl)−1,2,3,4-tetrahydroquinoline-2,4-dicarboxylate	C14H14N2O6	vit. B - cofactor		+			+	+
Methyl (indole-3-yl)acetate	C11H11NO2	IAA related		+	+		+	
Tryptophan	C11H12N2O2	IAA precursor		+	+	F, R, G	+	
Indole_acetate	C10H9NO2	IAA		+		F		
2,3-dihydroxybenzoate	C7H5O4	siderophore building block		+		F		
3-4-dihydroxybenzoate	C7H6O4	siderophore building block			+			
PAI-1 (N-(3-oxododecanoyl)-L-homoserine lactone)	C16H27NO4	quorum-sensing	+	+	+			
AAI	C12H19NO4	quorum-sensing		+	+		+	
VAI-2	C12H21NO3	quorum-sensing		+	+			
VAI-1	C10H15NO4	quorum-sensing		+	+		+	+
HAI-1	C8H13NO4	quorum-sensing		+	+		+	
N-3-hydroxydecanoyl-L-homoserine lactone	C14H25NO4	quorum-sensing	+		+	J		
Porphobilinogen	C10H14N2O4	AA derivate		+	+		+	+
Tyrosine	C9H11NO3	AA		+	+	R, G	+	+
Arogenate	C10H13NO5	AA precursor		+	+	R	+	+
4-Hydroxy-phenylpyruvate	C9H8O4	AA precursor		+	+		+	+
Phenylalanine	C9H11NO2	AA		+		F, R, G	+	+
L-SDAP	C11H18N2O7	AA precursor		+	+			
2-Isopropylmaleate	C7H10O4	AA precursor		+	+	R	+	
Delta-piperideine-2-6-dicarboxylate	C7H9NO4	AA precursor		+	+		+	+
O-Acetyl-L-homoserine	C6H11NO4	AA derivate		+	+		+	
Histidine	C6H9N3O2	AA		+	+	G, J		
Miraxanthin V	C17H18N2O6	betaxanthine			+		+	+
L-Dihydroxy-phenylalanine	C9H11NO4	betaxanthine			+		+	+
Glutathione	C10H17N3O6S	defense		+	+	R		
Tropodithietic acid	C8H4O3S2	antibiotic	+		+			
Inosine	C10H12N4O5	purin metabolism		+	+	F, R		
Thymidine	C10H14N2O5			+		R	+	+
Deoxycytidine	C9H13N3O4			+	+			
Phenylacetylcarbinol	C9H10O2				+			+
S-Methyl phenylethanethioate	C9H10OS				+	T		

*F, Fiore et al. (2015); G, Garcia et al. (2015); J, Johnson et al. (2016); R, Romano et al. (2014); T, Thiel et al. (2010). Garcia et al. inferred the metabolites in cocultures on the basis of genomic information. +, Detected; AA, amino acid; IAA, indole acetic acid*.

**Figure 5:**
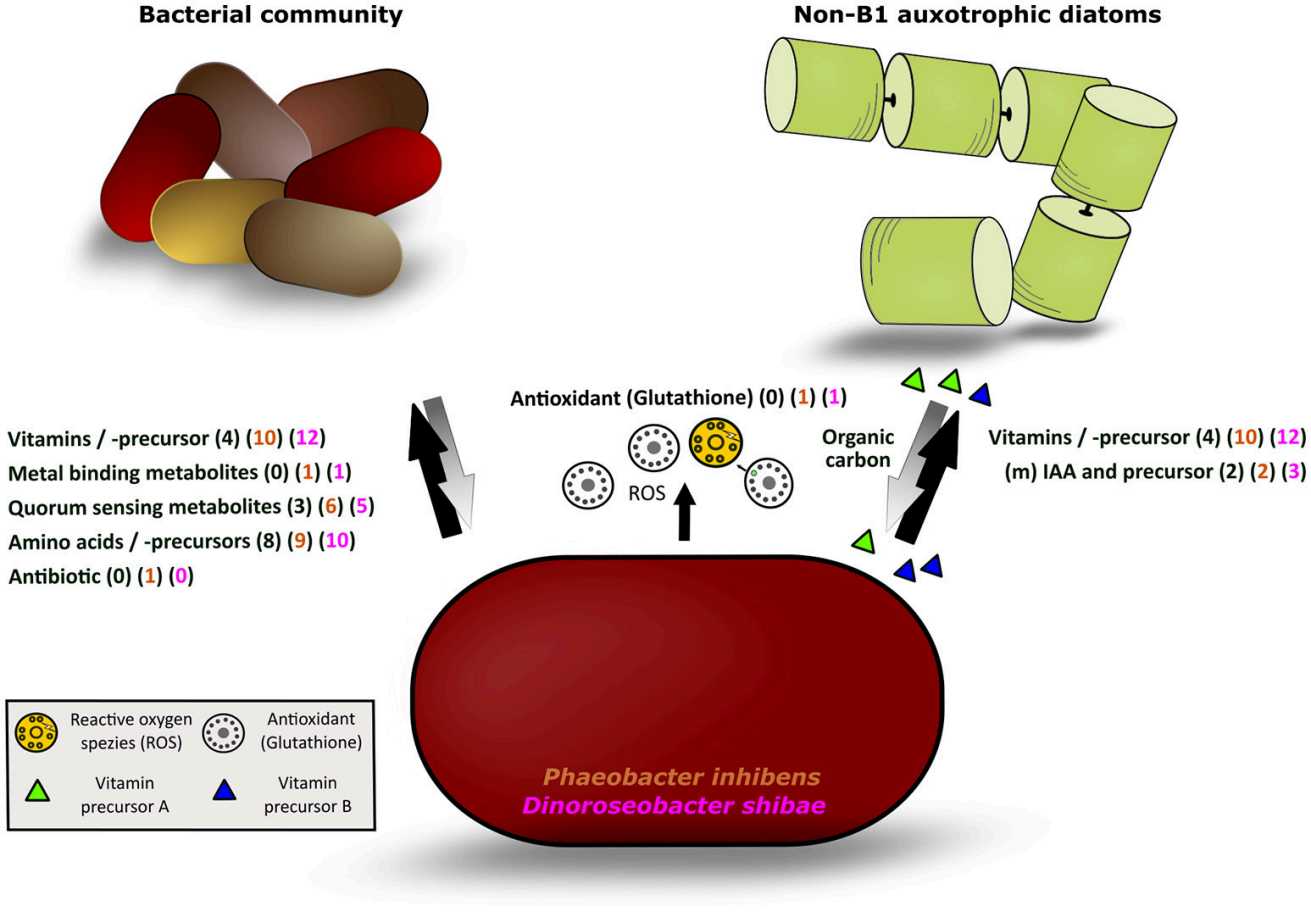
Illustration of the different classes of exometabolites released by *P. inhibens* and *D. shibae* and mutualisms with other bacteria and phytoplankton algae. Black arrow: detected metabolites released by both strains; gray arrow: potential response of the bacterial or phytoplankton communities to the bacteria releasing metabolites; in parentheses, the number of exometabolites of each class detected in the natural phytoplankton bloom (black) and released by *P. inhibens* (brown) and *D. shibae* (pink) are given.

The detailed analysis showed that 12 (34%) and 10 (28%) of the annotated compounds linked to metabolic pathways of *D. shibae* and *P. inhibens*, respectively, were B vitamins and/or late precursors of biosynthetic pathways of vitamins B_1_, B_2_, B_5_, B_6_, B_7_, and B_12_ (Figure 5, Table 1). Five (14%) and 6 (18%) of the annotated compounds were putatively quorum sensing-related metabolites and 10 (29%) and 9 (25%) amino acids and precursors of their biosynthetic pathways (Figure 5). Methyl-(indole-3-yl) acetate and tryptophan as precursors in the biosynthetic pathways of IAA were annotated as exometabolites of both organisms whereas IAA was annotated only in that of *D. shibae*. Altogether, the complete set of annotated compounds was linked to 22 and 20 biosynthetic pathways in *D. shibae* and in *P. inhibens* respectively (Table 1).

To further validate the compounds annotated by the genome-based metabolite prediction approach, the respective molecular masses were isolated and fragmented via positive charge collision in the FT-ICR-MS. We confirmed the presence of 7 metabolites in the exometabolomes of *D. shibae* and *P. inhibens* respectively, including vitamins, vitamin precursors, acylated homoserine lactones (autoinducers) for quorum sensing and TDA (Table 1). Support for the correct annotation of the compounds was also provided by their detection in other studies applying similar experimental approaches (Table 1). Several identical metabolites were detected by Fiore et al. (2015), Johnson et al. (2016), annotated by Romano et al. (2014), or predicted to be needed exogenously because of lacking biosynthetic pathways in annotated bacterial genomes (Garcia et al., 2015).

### Growth of diatoms on vitamin B1 and precursors

**Figure 6:**
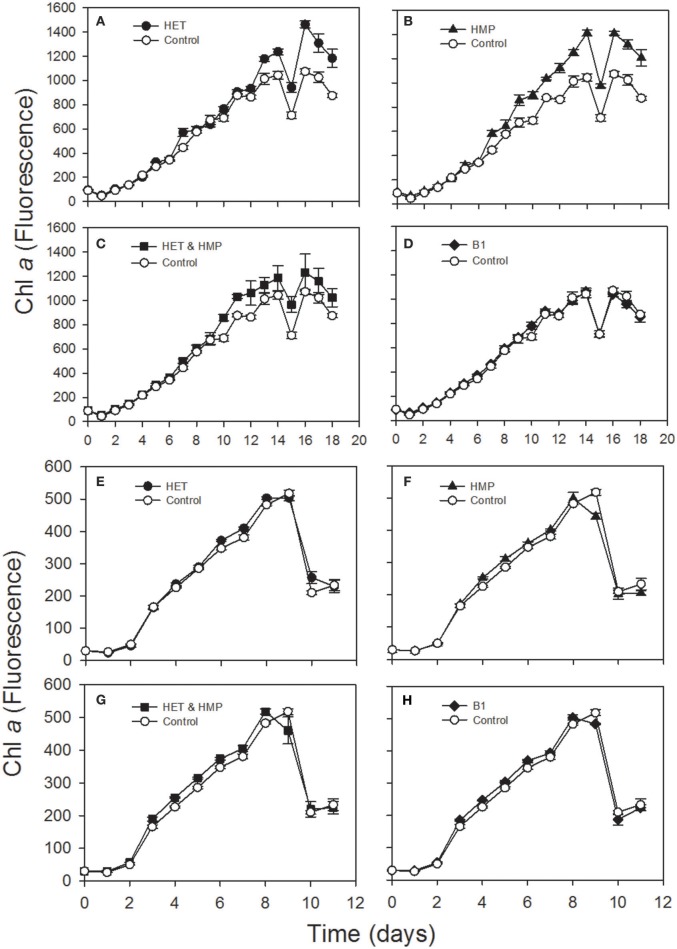
Chlorophyll *a* fluorescence over time of an axenic culture of the diatoms *Leptocylindrus danicus* **(A–D)** and Thalassiosira pseudonana **(E–H)** supplemented with 4-methyl-5-(β-hydroxyethyl)thiazole (HET; **A,E**), 4-amino-5-hydroxymethyl-2-methylpyrimidine (HMP; **B,F**), HET & HMP **(C,G)** and vitamin B_1_ **(D,H)** and a control without any of these supplements.

In order to test the second part of our hypothesis whether identified precursors indeed enhance growth of prototrophic algae we selected two diatoms, *Thalassiosira pseudonana* and *Leptocylindrus danicus* and grew them with the supplementation of HET, HMP, HET, and HMP or B_1_ and other vitamins they require (B_7_, B_12_). Supplementation of HET and HMP resulted in statistically significantly higher growth of both diatoms (*p* < 0.01; Student's *t*-test; Table S7). The growth rate and yield of *L. danicus* was enhanced by 17 and 35%, respectively, relative to a control, whereas the growth rate of *T. pseudonana* was enhanced by 22% but growth yield remained unaffected (Figure 6, Table S7). In both diatom cultures the growth stimulation differed among the various growth phases. The addition of vitamin B_1_, surprisingly, resulted in a lower growth stimulation of both diatoms than that of the precursors and only for *T. pseudonana* the growth rate was significantly higher than that of the control (Figure 6, Table S7).

### Identified exometabolites in marine DOM

To examine whether exometabolites produced by the two model strains of the *Roseobacter* group also occur in natural or naturally-derived DOM, we screened samples of a mesocosm experiment and from phytoplankton blooms in the North Sea. In the DOM of the mesocosm experiment in which a phytoplankton bloom was induced and various bacteria of the *Roseobacter* group were present (Osterholz et al., 2015), 19 MF were detected which matched those found also in the cultures of both strains. Sixteen and 15 MF were attributed to the cultures of *P. inhibens* and *D. shibae* respectively, and 14 occurred in both cultures. Eight MF included amino acids or biosynthetic precursors, three late biosynthetic precursors of vitamins and cofactors, three were autoinducers of quorum sensing and two the plant auxin IAA and its inactive form methyl-IAA (Table 1). In samples from in and outside phytoplankton blooms in the North Sea, 15 MF matched those found in the exometabolomes of both strains. Four of those were biosynthetic precursors of vitamins and cofactors including those detected in the mesocosm experiment, six were amino acids or biosynthetic precursors, matching those detected in the mesocosm experiment, and one was an autoinducer to quorum sensing found also in the mesocosm experiment (Table 1).

## Discussion

Our exometabolome analyses are based predominantly on an untargeted approach using FT-ICR-MS, which enables an ultrahigh resolution of individual molecular masses with a relative but no absolute quantification. The results show that *P. inhibens* and *D. shibae* both exhibit distinct exometabolite patterns consisting of 2,767 and 3,354 distinct MF and variations as a function of the carbon source. We were able to identify 2.3 and 2.6% of the exometabolites of *P. inhibens* and *D. shibae*, respectively, by comparing detected MF and genome-predicted metabolites. The observation that more than 97% of the MF consist of unknown chemical compounds, also reported in previous studies (Romano et al., 2014; Fiore et al., 2015; Johnson et al., 2016), is surprising and illustrates that the majority of MF comprises compounds is not predicted by genome annotated metabolites. There are indications that some of these compounds are metabolic waste (Fiore et al., 2015) but presumably other biological and physico-chemical reactions contribute to the formation of these compounds. The diversity of the exometabolome and implications for a better understanding of the bacterial processing of organic matter to shape the marine DOM pool are discussed elsewhere (Noriega-Ortega et al., in preparation). Here we focus on the identified exometabolites and the implications these findings have for the understanding of mutualistic interactions among bacteria and algae in marine ecosystems. The detection of seven exometabolites was further supported by fragmentation and we did not find a single case of mismatching fragments. We obtained further support of the correct MF by the genome-based exometabolite prediction. Hence our MF assignment is based on three independent methods and thus appears to be a solid base for discussing their significance in the interplay among marine microbes.

### Significance of identified exometabolites

In both strains we identified MF identical to late biosynthetic precursors of and/or the vitamins B_1_, B_2_, B_5_, B_6_, B_7_, and B_12_, IAA and its methylated form, metal-acquisition growth factors, autoinducers for quorum sensing and biosynthetic precursors of several amino acids. Pyridoxal, the dephosphorylated form of vitamin B_6_, alpha-ribazole, methyl-IAA and 3 autoinducers were also putatively detected in the DOM of a naturally-derived phytoplankton bloom (Osterholz et al., 2015) and the same vitamin precursors, alpha-ribazole-5 phosphate and one of the autoinducers also in the DOM of a North Sea phytoplankton bloom. Most MF identical to biosynthetic precursors of amino acids were also detected in the phytoplankton bloom samples. On the basis of genomic information of both strains and previous data, the vitamins and autoinducers were expected to be present in the exometabolome (Newton et al., 2010; Wagner-Döbler et al., 2010; Thole et al., 2012). However, the putative detection of late precursors of all B vitamins except one, methyl-IAA and five biosynthetic precursors of amino acids in the exometabolome of each strain and in the DOM of the phytoplankton bloom samples was surprising. It showed that these strains and presumably other microorganisms as well release a much greater variety of exometabolites than expected. Even though previous studies found a few of the precursors we detected, such a rich bouquet of biosynthetic precursors has neither been reported before in any bacterial exometabolome nor in the DOM of phytoplankton blooms.

The B_2_ precursor dimethyl-D-ribityl-lumazine and/or the B_12_ precursor alpha-ribazole and two amino acid precursors were detected as exometabolites of a *Pseudovibrio* strain and *Ruegeria pomeroyi* (Romano et al., 2014; Johnson et al., 2016). The final precursor of the thiazole moiety of vitamin B_1_, HET, we detected in both strains, is known to be used by several green algae, cryptophytes and dinoflagellates instead of B_1_ (Lwoff, 1947; Droop, 1958; Turner, 1979). The pyrimidine moiety of vitamin B_1_ which we did not detect, HMP, has been reported to be released by cyanobacteria, a marine betaproteobacterium and the alga *Dunaliella tertiolecta* (Carini et al., 2014). Results of previous research provides evidence that HMP and HET can be used in a salvage pathway for biosynthesis of thiamine by various B_1_-auxotrophic eukaryotic algae and by Pelagibacterales (Turner, 1979; Carini et al., 2014; McRose et al., 2014; Paerl et al., 2015). There is most recent evidence, however, that HMP and unknown HET-related precursors can support growth of B_1_-auxotrophic microeukaryotic marine algae via various bacteria and that these precursors are present in the open ocean (Paerl et al., 2017). Our results of the growth experiments with *T. pseudonana*, and *L. danicus* indicate that HMP and HET also stimulate growth of these vitamin B_1_ prototrophic coastal diatoms. It was unexpected that the effect was even higher than that of the addition of B_1_. This result is surprising and implies that *L. danicus* either lacks a vitamin B_1_ transporter and/or that in both diatoms these precursors do not only compensate the lacking genetic capabilities of auxotrophic microbes but that HET and HMP have a so far unknown growth-promoting effect on vitamin B_1_ prototrophic diatoms. If this stimulatory effect on vitamin B_1_ prototrophic organisms is also true for other microorganisms the release of these precursors has even greater implications for controlling growth of these planktonic communities.

Pantoate as a precursor of vitamin B_5_ was detected in the exometabolome of *D. shibae*. It is unknown whether this B_5_ precursor can be used by marine algae or bacteria. An uptake system for pantoate, however, has been described for *Salmonella enterica* (Ernst and Downs, 2015) and thus is likely to exist in marine bacteria enabling them to use this precursor. Pyridoxal as the dephosphorylated form of vitamin B_6_ was detected in the exometabolome of both strains and in the DOM of both phytoplankton blooms. Pyridoxal kinase (EC 2.7.1.35), the enzyme to phosphorylate this inactive form of vitamin B_6_, is encoded in the genome of many bacteria as documented by a genomic search (https://img.jgi.doe.gov/cgi-bin/mer/main.cgi). If microbes can take up pyridoxal like *Sacharomyces cerevisiae* (Stolz and Vielreicher, 2003), this inactive form may be a so far neglected source of vitamin B_6_.

Dethiobiotin, the last stage in the biosynthesis of vitamin B_7_ (biotin), was detected in the exo-metabolome of *P. inhibens*. Extracellularly provided dethiobiotin has been demonstrated to cause diverging effects. Exogenous simultaneous supply of biotin and dethiobiotin caused a growth inhibition in several biotin-requiring fungi and bacteria including *Sacharomyces, Sordaria* and *Lactobacilli*, and was termed the anti-biotin effect (Dittmer et al., 1944; Lilly and Leonian, 1944). Contrary observations were reported for other fungi and Lactobacilli and a freshwater cyanobacterium, in which biotin auxotrophy was compensated by dethiobiotin addition (Dittmer et al., 1944; Lilly and Leonian, 1944; Bowman and DeMoll, 1993). Hence the significance of released dethiobiotin in marine microbial communities including the mycoplankton is still open and needs further studies.

Alpha-ribazole, a lower ligand building block of vitamin B_12_, was released by both strains and detected in the phytoplankton bloom samples. The bioactive corrinoid cofactor vitamin B_12_ has been shown to be a controlling factor of primary production in pelagic ecosystems (Bertrand et al., 2007; Koch et al., 2011). Its cofactor binding, function and catalysis highly depends on the attached lower ligand (Lengyel et al., 1960; Stupperich et al., 1988; Renz, 1999; Yi et al., 2012). It has been shown, however, that the genome of *Listeria innocua*, lacking the genes for alpha-ribazole biosynthesis, encodes a transporter system of alpha-ribazole, cblT, and is able to synthesize B_12_ when this moiety is available as an exogenous source (Gray and Escalante-Semerena, 2010). Remodeling of corrinoids, such as vitamin B_12_, by complementation of the lower ligand via uptake of exogenous compounds appears to be a common phenomenon within microbial communities (Gray and Escalante-Semerena, 2010; Keller et al., 2014; Men et al., 2014). Available 5,6-dimethylbenzimidazole (DMB), an alpha-ribazole precursor, allows major phytoplankton groups to remodel pseudocobalamin, commonly produced by cyanobacteria, into a usable corrinoid cofactor structure (Helliwell et al., 2016). Our results indicate that corrinoid building blocks are exchanged in marine ecosystems and that roseobacters such as *D. shibae* and *P. inhibens* may be providers of the most common and bioactive corrinoid cofactor lower ligand.

The auxin IAA has recently been identified to be secreted by various freshwater and marine bacteria and to be an important growth factor of various green algae and a diatom (Bagwell et al., 2014; Zhang et al., 2014; Amin et al., 2015, Fiore et al., 2015). It was also found in a phytoplankton bloom in the Pacific, in a eutrophic lake dominated by cyanobacteria and to be produced by *P. inhibens* (Zhang et al., 2014; Amin et al., 2015; Segev et al., 2016). We detected IAA in the exometabolome of *D. shibae* and its precursor tryptophan in the exometabolome of both strains and the naturally derived phytoplankton bloom. Tryptophan has been shown to enhance the production of IAA in *P. inhibens* (Segev et al., 2016) and also in the haptophyte *Emiliania huxleyi* (Labeeuw et al., 2016). It thus appears to be important in controlling the production of IAA in these roseobacters and the haptophyte. In both strains and in the naturally derived phytoplankton bloom, we also detected methyl-IAA, a related compound which was neither detected previously in the exometabolome of marine bacteria nor in marine DOM samples. It has been shown in *Arabidopsis* that methyl-IAA is an inactive form which can be taken up but needs to be demethylated by an esterase to generate the active IAA (Yang et al., 2008). Because of the more hydrophobic form of methyl-IAA as compared to IAA, these authors suggest that it is more easily transported across the cell membrane, possibly even diffuses, thus enhancing the exploitation of the exogenous supply with the subsequent need to demethylate it intracellularly. Another aspect of hydrophobic compounds released into the water is that enhanced hydrophobicity results in a faster supply through this hydrophilic medium thus reducing the time of action and enhancing the accumulation at the target site, e.g. the cell surface (Maier et al., 1994). Hence it is conceivable that the methylated form of IAA in pelagic ecosystems leads to a more efficient use of this important auxin by phytoplankton.

The metal-binding 2,3-dihydroxybenzoate was detected in the exometabolome of *D. shibae*. This building block of the siderophore enterobactin is known to be secreted by heterotrophic and cyanobacteria under iron-limiting conditions (Young et al., 1967; Byers and Lankford, 1968; Fiore et al., 2015) and to enhance the expression of the 2,3-dihydroxybenzoate-AMP ligase, catalyzing an essential synthesis step toward the formation of enterobactin (Khalil and Pawelek, 2011). Other strains of the *Roseobacter* group are known to secrete enterobactin but not 2,3-dihydrobenzoate (Hogle et al., 2016). Hence, *D. shibae* appears to have the potential to provide 2,3-dihydroxybenzoate to marine bacterioplankton communities, thus inducing enterobactin synthesis and favoring iron uptake by itself and other bacteria. A building block of the siderophore petrobactin, 3,4-dihydroxybenzoate, was detected in the exometabolome of *P. inhibens* and not found before as a bacterial exometabolite. It has been shown that *P. inhibens* is able to produce enterobactin (Thole et al., 2012). Thus, 3,4-dihydroxybenzoate may have a similar role in metal acquisition as 2,3-dihydroxybenzoate (see above).

Glutathione was detected in the exometabolome of both strains and previously found also in that of a *Pseudovibrio* strain (Romano et al., 2014). Besides essential intracellular functions, glutathione is a fundamental extracellular protectant for bacteria, in particular as an antioxidant when reactive oxygen species (ROS) are present (Smirnova and Oktyabrsky, 2005; Montoya, 2013; Smirnova et al., 2015). One reason for the presence of glutathione in the exometabolome may be stress caused by the growth conditions and high cell densities and the possible protection against ROS. On the other hand, glutathione has been found in nanomolar concentrations in the oligotrophic north Pacific (Dupont et al., 2006) even though it is rapidly photooxidized (Moingt et al., 2010). Therefore, it is conceivable that bacteria such as our model strains actively excrete glutathione for protection against ROS produced by photochemical DOM oxidation.

Biosynthetic precursors of amino acids were previously reported in bacterial exometabolomes but interpreted as a result of an overflow metabolism (Paczia et al., 2012; Romano et al., 2014). As we found these precursors also in the DOM of phytoplankton blooms, we suggest that they are either actively secreted or released by dividing and growing cells or due to mortality by grazing or viral lysis of bacterial communities and are not a result of an overflow metabolism. They can potentially be used for amino acid biosynthesis by other bacteria but this pathway has yet to be shown. In support of this suggestion, it has recently been shown that bacterial mutants, missing biosynthetic genes in amino acid pathways, have a growth benefit over the wild type when supplied with the respective precursors or amino acids (D'Souza et al., 2014; Waschina et al., 2016). These authors consider amino acid cross-feeding as a specialized evolutionary mechanism of how bacterial subpopulations can receive mutual benefits by saving biosynthetic costs. Auxotrophy of essential amino acids can potentially be conquered by public amino acid goods and even facilitate metabolic interdependency or symbiosis (McCutcheon and Moran, 2007; Garcia et al., 2015). Our findings of several essential and non-essential amino acids and respective biosynthetic precursors in the exometabolome of both strains and in the DOM of the phytoplankton bloom samples suggests that beneficial amino acid cross-feeding also occurs in marine microbial communities.

### The exometabolome: a market place of microbial metabolites

The release of a wealth of exometabolites by the two model strains indicates that both of them may function as important suppliers of growth factors as well as of biosynthetic precursors, so-called public goods (Morris et al., 2012), to other pro- and eukaryotes in marine ecosystems. *Phaeobacter inhibens* dwells in biofilms (Gram et al., 2015) and both model strains live in association with microalgae and pelagic environments (Wagner-Döbler et al., 2010; Gifford et al., 2014; Segev et al., 2016) thus suggesting that these exometabolites are released as public goods in biofilm-associated as well as in pelagic marine communities. Biofilms with dense colonization of diverse bacterial communities may include surface-associated habitats but also marine aggregates which often form during phytoplankton blooms (Simon et al., 2002). Bacteria releasing public goods were recently termed Black Queen in the context of the Black Queen hypothesis (BQH; Morris et al., 2012; Morris, 2015), a scenario in which other bacteria benefit from losing genetic traits to synthesize certain growth factors such as vitamins or parts of their biosynthetic pathways when exogenous supply of these compounds is consistently available. Aggregate-associated bacteria acting as Black Queens may also supply free-living pelagic bacteria and phytoplankton algae in the surrounding water with public goods. Genome streamlining features were reported from various pelagic bacteria including *Pelagibacterales*, the SAR86 clade and *Prochlorococcus* (Dupont et al., 2012; Carini et al., 2014; Giovannoni et al., 2014) but also from free-living and symbiotic bacteria dwelling in nutrient-rich or constant environments (Van de Guchte et al., 2006; McCutcheon and Moran, 2007; D'Souza et al., 2014). Supply of released compounds as public goods to other microbes is part of a complex network of microbial interactions and there are quite a few public goods in this context, but also private metabolic goods not shared, which act beyond the concept of the BQH (Morris, 2015; Estrela et al., 2016). Our results in fact indicate that the two vitamin B_1_ prototrophic diatoms benefit from supply by the B_1_ precursors HET and HMP, a scenario not considered by the BQH. It must also be kept in mind that in a microbial community mutual interactions exist between the primary producers secreting substrates to the (photo)heterotrophic microbes and that different microbes are distinct in their capabilities, e.g., in hydrolyzing polymers. For instance *Flavobacteria* exhibit a wealth of polysaccharide hydrolyzing enzymes (Teeling et al., 2012) whereas roseobacters are very limited in these polymer-degrading traits (Hahnke et al., 2013). Further, it has been shown that different bacteria, each missing distinct genomic metabolic traits and exhibiting streamlined genomic features, coexist by complementing each other with metabolites for which they are auxotrophic (Garcia et al., 2015). A microbial community with mutual interdependencies between two or among a group of microbes and no unidirectional flows of public and private goods among various bacteria and other microbes appears to be a more suitable model to describe this complex network, a marketplace of microbial metabolites (Figure 5; Zelezniak et al., 2015). Such scenarios presumably characterize the dynamic ecosystems in which both model strains dwell: In tighter or looser association with algae and other bacteria on biofilms or during phytoplankton blooms. Both scenarios exhibit many mutual interactions among a multitude of organisms with non-streamlined as well as streamlined genomes.

## Author contributions

All authors designed the experiments. GW and BN carried out the experiments, GW identified the exometabolites and wrote the first draft of the manuscript. MS and GW wrote the final version of the manuscript. All authors critically reviewed and added aspects to the manuscript.

### Conflict of interest statement

The authors declare that the research was conducted in the absence of any commercial or financial relationships that could be construed as a potential conflict of interest.
