# Supplementary material for: The Exometabolome of Two Model Strains of the Roseobacter Group: A Marketplace of Microbial Metabolites
Source: Front Microbiol. 2017 Oct 12;8:1985. doi: 10.3389/fmicb.2017.01985 (PMC5643483; doi:10.3389/fmicb.2017.01985)

Supplementary information to:

The exometabolome of two model strains of the *Roseobacter* group: A marketplace of microbial metabolites

Gerrit Wienhausen, Beatriz Ortega-Noriega, Jutta Niggemann, Thorsten Dittmar, Meinhard Simon*

Institute for Chemistry and Biology of the Marine Environment (ICBM),

University of Oldenburg, Germany,

Carl-von-Ossietzky-Strasse 9-11,

D-26129 Oldenburg, Germany

*Correspondence: Meinhard Simon:

E-mail: m.simon@icbm.de

Supplementary Table S1: Mol% of detected amino acids in the exometabolome of *P. inhibens* and *D. shibae*

Supplementary Table S2: Mol% of detected monosaccharides in the exometabolome of *P. inhibens* and *D. shibae*

Supplementary Table S3: List of all detected masses and assigned formulas of the exometabolome of *D. shibae* grown on the three carbon sources. (see Table on extra Excel sheet)

Supplementary Table S4: List of all detected masses and assigned formulas of the exometabolome of *P. inhibens* grown on the three carbon sources. (see Table on extra Excel sheet)

Supplementary Table S5: Exometabolites of degradation pathways of *P. inhibens* and *D. shibae*

Supplementary Table S6: Exometabolites of spontaneous non-enzymatic chemical-reactions of *P. inhibens* and *D. shibae*

Supplementary Table S7: Growth rate and yield of *Leptocylindrus danicus* and *Thalassiosira pseudonana* amended with precursors and vitamin B1

Supplementary Figure S1: Cell numbers of *P. inhibens* and *D. shibae* during growth experiments

Supplementary Table S1:

Mol% (± standard deviation) of detected amino acids in dissolved combined amino acids (DCAA) in the exometabolome of *P. inhibens* and *D. shibae* grown on glutamate, glucose and acetate or succinate, respectively, during the entire growth phases. In the treatment with glutamate as C source DCAA could not be determined (nd) by HPLC until the stationary phase because the large glutamate peak interfered with the HPLC analysis.

C source	Time	Growth phase	Mol%													
	(h)		Asp	Glu	His	Ser	Arg	Gly	Thre	ß-Ala	Ala	Try	Val	Phe	Ile	Leu
P. inhibens																
Glutamate	0	inoculation	nd	nd	nd	nd	nd	nd	nd	nd	nd	nd	nd	nd	nd	nd
	8	lag	nd	nd	nd	nd	nd	nd	nd	nd	nd	nd	nd	nd	nd	nd
	14	exponential	nd	nd	nd	nd	nd	nd	nd	nd	nd	nd	nd	nd	nd	nd
	22	stationary	12,4 (± 1,0)	10,1 (± 2,8)	1,9 (± 0,1)	5,8 (± 0,6)	2,4 (± 0,2)	20,2 (± 1,3)	10,4 (± 0,9)	0,3 (± 0,0)	13,7 (± 1,0)	2,4 (± 0,2)	5,7 (± 0,6)	2,7 (± 0,2)	3,6 (± 0,4)	8,4 (± 0,7)
Glucose	0	inoculation	0,0 (± 0,0)	83,8 (± 0,5)	0,0 (± 0,0)	0,0 (± 0,0)	0,0 (± 0,0)	0,0 (± 0,0)	0,0 (± 0,0)	0,0 (± 0,0)	3,7 (± 0,6)	0,0 (± 0,0)	12,6 (± 0,5)	0,0 (± 0,0)	0,0 (± 0,0)	0,0 (± 0,0)
	16	lag	0,0 (± 0,0)	0,0 (± 0,0)	0,0 (± 0,0)	0,0 (± 0,0)	0,1 (± 0,0)	0,8 (± 0,1)	4,2 (± 0,6)	0,0 (± 0,0)	55,9 (± 2,4)	1,1 (± 0,2)	38,0 (± 0,8)	0,0 (± 0,0)	0,0 (± 0,0)	0,0 (± 0,0)
	40	exponential	12,2 (± 0,0)	22,5 (± 0,0)	1,5 (± 0,2)	1,9 (± 0,5)	5,2 (± 0,0)	19,6 (± 0,2)	6,9 (± 0,0)	0,5 (± 0,0)	13,4 (± 0,0)	4,1 (± 0,0)	8,6 (± 1,8)	1,9 (± 0,1)	0,6 (± 0,7)	1,0 (± 0,0)
	62	stationary	13,2 (± 0,0)	20,0 (± 0,2)	2,0 (± 0,0)	4,6 (± 0,0)	5,1 (± 0,1)	21,7 (± 0,1)	6,7 (± 0,0)	0,6 (± 0,0)	11,4 (± 0,0)	4,2 (± 0,0)	4,3 (± 0,0)	2,5 (± 0,0)	1,0 (± 0,1)	2,6 (± 0,3)
Acetate	0	inoculation	0,0 (± 0,0)	0,0 (± 0,0)	0,0 (± 0,0)	0,0 (± 0,0)	0,0 (± 0,0)	0,0 (± 0,0)	0,0 (± 0,0)	0,0 (± 0,0)	12,5 (± 0,1)	0,0 (± 0,0)	87,5 (± 0,4)	0,0 (± 0,0)	0,0 (± 0,0)	0,0 (± 0,0)
	32	lag	20,4 (± 0,2)	58,0 (± 1,0)	0,0 (± 0,0)	0,0 (± 0,0)	3,9 (± 0,3)	3,3 (± 1,0)	6,9 (± 0,5)	0,0 (± 0,0)	7,5 (± 0,4)	0,0 (± 0,0)	0,0 (± 0,0)	0,0 (± 0,0)	0,0 (± 0,0)	0,0 (± 0,0)
	52	exponential	20,8 (± 4,0)	12,5 (± 1,4)	0,6 (± 0,6)	4,8 (± 2,3)	4,5 (± 0,7)	22,4 (± 4,0)	8,1 (± 2,4)	0,3 (± 0,0)	9,3 (± 1,6)	2,3 (± 0,6)	4,2 (± 1,6)	1,9 (± 1,0)	2,4 (± 1,7)	6,0 (± 3,2)
	68	stationary	21,4 (± 41,8)	9,4 (± 12,8)	1,0 (± 0,4)	6,8 (± 9,9)	3,0 (± 2,4)	21,9 (± 37,1)	8,8 (± 19,7)	0,1 (± 0,3)	8,5 (± 12,2)	2,1 (± 2,6)	4,4 (± 4,1)	2,5 (± 3,2)	2,8 (± 2,4)	7,3 (± 8,5)
D. shibae																
Glutamate	0	inoculation	nd	nd	nd	nd	nd	nd	nd	nd	nd	nd	nd	nd	nd	nd
	15	lag	nd	nd	nd	nd	nd	nd	nd	nd	nd	nd	nd	nd	nd	nd
	31	exponential	nd	nd	nd	nd	nd	nd	nd	nd	nd	nd	nd	nd	nd	nd
	45	stationary	0,5 (± 0,2)	33,8 (± 0,7)	0,6 (± 0,0)	2,5 (± 0,1)	0,4 (± 0,1)	16,3 (± 0,0)	1,6 (± 0,1)	0,0 (± 0,0)	26,4 (± 0,1)	0,0 (± 0,0)	4,7 (± 0,3)	0,8 (± 0,5)	0,0 (± 0,0)	12,4 (± 0,1)
Glucose	0	inoculation	2,9 (± 0,0)	7,7 (± 0,7)	10,4 (± 0,0)	10,2 (± 0,3)	8,1 (± 0,3)	13,7 (± 0,1)	9,0 (± 0,0)	3,9 (± 2,0)	10,4 (± 0,0)	8,6 (± 0,0)	6,5 (± 0,0)	5,9 (± 0,0)	2,5 (± 1,3)	0,0 (± 0,0)
	17	lag	3,9 (± 0,5)	13,5 (± 0,7)	5,1 (± 0,0)	5,3 (± 0,1)	4,1 (± 0,1)	15,1 (± 0,4)	6,3 (± 0,2)	5,1 (± 0,0)	20,2 (± 1,0)	5,0 (± 0,1)	4,7 (± 0,2)	4,3 (± 0,1)	4,2 (± 0,0)	3,2 (± 1,2)
	35	exponential	6,7 (± 0,3)	14,9 (± 0,7)	3,3 (± 0,0)	6,1 (± 0,6)	4,0 (± 0,2)	15,2 (± 1,5)	5,5 (± 0,1)	3,4 (± 0,0)	19,5 (± 0,3)	4,0 (± 0,1)	4,7 (± 0,1)	3,7 (± 0,2)	3,3 (± 0,1)	5,7 (± 0,2)
	75	stationary	7,4 (± 0,3)	15,7 (± 0,3)	2,2 (± 0,0)	5,5 (± 0,1)	3,7 (± 0,0)	15,4 (± 0,5)	5,1 (± 0,2)	1,6 (± 0,0)	22,2 (± 0,3)	3,3 (± 0,1)	5,3 (± 0,2)	4,7 (± 0,1)	2,8 (± 0,1)	5,0 (± 0,2)
Succinate	0	inoculation	0,0 (± 0,0)	0,0 (± 0,0)	0,0 (± 0,0)	0,0 ± 0,0)	0,0 (± 0,0)	8,5 (± 3,7)	2,7 (± 0,0)	1,4 (± 0,0)	0,0 (± 0,0)	0,0 (± 0,0)	43,1 (± 0,0)	26,4 (± 0,4)	0,0 (± 0,0)	18,0 (± 0,7)
	9	lag	0,0 (± 0,0)	9,7 (± 52,8)	0,0 (± 0,0)	6,5 (± 1,3)	1,3 (± 0,3)	29,6 (± 0,5)	6,7 (± 0,9)	0,1 (± 0,0)	11,3 (± 1,7)	0,8 (± 0,2)	34,0 (± 6,8)	0,0 (± 0,0)	0,0 (± 0,0)	0,0 (± 0,0)
	19	exponential	4,8 (± 0,2)	23,0 (± 66,9)	0,5 (± 1,2)	4,6 (± 0,7)	4,4 (± 4,4)	24,1 (± 0,1)	7,2 (± 0,0)	0,6 (± 0,2)	18,4 (± 0,0)	1,4 (± 0,0)	3,5 (± 0,1)	2,5 (± 0,1)	1,0 (± 0,0)	4,1 (± 0,2)
	25	stationary	6,5 (± 0,1)	21,6 (± 6,2)	0,3 (± 0,0)	4,1 (± 0,1)	2,3 (± 0,3)	20,6 (± 0,6)	6,0 (± 0,1)	0,4 (± 0,2)	20,3 (± 0,2)	1,7 (± 0,0)	4,4 (± 0,1)	3,1 (± 0,1)	2,3 (± 0,1)	6,2 (± 0,4)

Supplementary Table S2:

Mol% (± standard deviation) of detected monosaccharides in dissolved combined neutral monosaccharides (DCNCHO) in the exometabolome of *P. inhibens* and *D. shibae* grown on glutamate, glucose and acetate or succinate, respectively, during the entire growth phases. In the treatment with glucose as C source DCNCHO could not be determined (nd) by HPLC until the stationary phase because the large glucose peak interfered with the HPLC analysis.

C source	Time (h)	Growth phase	Mol% Fucose	Rhamnose	Galactose	Glucose
P. inhibens						
Glutamate	0.0	inoculation	0,0 (± 0,0)	0,0 (± 0,0)	0,0 (± 0,0)	0,0 (± 0,0)
	8.0	lag	10,2 (± 0,0)	6,6 (± 0,0)	5,9 (± 0,0)	77,3 (± 0,3)
	14.0	exponential	8,6 (± 0,0)	6,5 (± 0,0)	5,0 (± 0,0)	79,8 (± 0,3)
	22.0	stationary	7,8 (± 0,0)	3,0 (± 0,0)	5,2 (± 0,0)	80,7 (± 0,1)
Glucose	0.0	inoculation	nd	nd	nd	nd
	16.0	lag	nd	nd	nd	nd
	40.0	exponential	nd	nd	nd	nd
	62.0	stationary	5,7 (± 1,9)	1,5 (± 1,5)	2,5 (± 0,6)	90,2 (± 122,6)
Acetate	0.0	inoculation	0,0 (± 0,0)	0,0 (± 0,0)	0,0 (± 0,0)	0,0 (± 0,0)
	32.0	lag	21,5 (± 0,0)	11,5 (± 0,3)	8,1 (± 0,0)	58,9 (± 0,2)
	52.0	exponential	20,7 (± 0,0)	13,0 (± 0,1)	11,8 (± 0,2)	54,6 (± 0,0)
	68.0	stationary	17,8 (± 0,2)	12,9 (± 0,2)	11,0 (± 0,2)	58,3 (± 0,9)
D. shibae						
Glutamate	0	inoculation	0,0 (± 0,0)	0,0 (± 0,0)	0,0 (± 0,0)	0,0 (± 0,0)
	15	lag	0,0 (± 0,0)	33,3 (± 0,7)	28,6 (± 0,7)	38,1 (± 0,5)
	31	exponential	0,0 (± 0,0)	24,7 (± 0,4)	49,3 (± 0,7)	25,9 (± 0,4)
	45	stationary	0,0 (± 0,0)	28,0 (± 1,0)	51,1 (± 1,9)	21,0 (± 0,8)
Glucose	0	inoculation	nd	nd	nd	nd
	17	lag	nd	nd	nd	nd
	35	exponential	nd	nd	nd	nd
	75	stationary	nd	nd	nd	nd
Succinate	0	inoculation	0,0 (± 0,0)	0,0 (± 0,0)	0,0 (± 0,0)	0,0 (± 0,0)
	9	lag	0,0 (± 0,0)	44,0 (± 0,3)	56,0 (± 0,1)	0,0 (± 0,0)
	19	exponential	0,0 (± 0,0)	35,6 (± 0,5)	47,6 (± 0,7)	16,8 (± 0,2)
	25	stationary	0,0 (± 0,0)	44,6 (± 2,2)	44,2 (± 2,2)	11,3 (± 0,5)

Table S5: Exometabolites originating from degradation pathways of *Phaeobacter inhibens* and *Dinorosebacter shibae* and their molecular formula.

Compound	Molecular Formula	Degradation /recycling	*P. inhibens*	*D. shibae*
L-kynurenine	C10H12N2O3	tryptophan degradation I	+	+
3-hydroxy-L-kynurenine	C10H12N2O4	tryptophan degradation to 2-amino-3-carboxymuconate semialdehyde	+	+
thymidine	C10H14N2O5	pyrimidine deoxyribonucleosides degradation	+	
N-formylkinurenine	C11H12N2O4	tryptophan degradation I	+	+
L-alanyl-gamma-D-glutamyl-meso-diaminopimelic acid	C15H26N4O8	anhydromuropeptides recycling	+	
3,4-dihydroxy-9,10-secoandrosta-1,3,5(10)-triene-9,17-dion	C19H24O4	androstenedione degradation	+	+
3-hydroxy-5917-trioxo-45910-Diseco A	C19H24O6	androstenedione degradation	+	+
di-homo-γ-linolenate	C20H34O2	fatty acid activation	+	
7,8-dihydrolumazine	C6H6N4O2	guaiacylglycerol-β-guaiacyl ether degradation	+	
4-imidazolone-5-	C6H8N2O3	histidine degradation	+	+
3-4-dihydroxybenzoate	C7H6O4	vanillin and vanillate degradation I	+	
deoxyuridine	C9H12N2O5	pyrimidine deoxyribonucleosides degradation	+	
uridine	C9H12N2O6	UTP and CTP dephosphorylation I	+	
deoxycytidine	C9H13N3O4	pyrimidine deoxyribonucleosides degradation	+	
4-hydroxy-2-nonenal	C9H16O2	conjugation with glutathione	+	
homogentisate	C8H8O4	tyrosine degradation I		+
1,6-anhydro-*N*-acetyl-β-muramate	C11H17NO7	anhydromuropeptides recycling		+
N-acetyl-β-D-glucosamine(anhydrous)-*N*-acetylmuramate	C19H30N2O12	anhydromuropeptides recycling		+
deoxyinosine	C10H12N4O4	purine deoxyribonucleosides degradation		+
2-trans-6-trans-farnesol	C15H26O	glycerophosphodiester degradation		+
3,4-dihydroxymandelate	C8H8O5	glycerophosphodiester degradation		+
3,4-dihydroxyphenylglycol	C8H10O4	glycerophosphodiester degradation		+
ferulic acid	C10H10O4	cellulose and hemicellulose degradation		+
Androst-4-ene	C19H26O2	androstenedione degradation		+

Table S6: Exometabolites assigned to spontaneous non-enzymatic chemical-reactions of *Phaeobacter inhibens* and *Dinorosebacter shibae* and their molecular formula.

**Spontaneous non-enzymatic chemical-reaction metabolites**	**Molecular Formula**	***P. inhibens***	***D. shibae***
4-methylumbelliferone	C10H8O3	+	+
scopoletine	C10H8O4	+	+
5-hydroxy-tryptophan	C11H12N2O3	+	
β-D-ribosylnicotinate	C11H13NO6	+	+
nicotinamide ribose	C11H14N2O5	+	+
lotaustralin	C11H19NO6	+	+
4-methylumbelliferyl glucoside	C16H18O8	+	+
scopolin	C16H18O9	+	+
estrone	C18H22O2	+	
3-beta-hydroxyandrost-5-en-17-one	C19H28O2	+	
beta-hydroxyandrost-5-en-17-one -3-sulfate	C19H30O5S	+	
3-ethylmalate	C6H10O5	+	+
3,4-dihydroxyphenylglycol	C8H10O4	+	
3-methoxy-4-hydroxyphenylglycolaldehyde	C9H10O4	+	+
isoquinolin-12H-one	C9H7NO	+	
hippurate	C9H9NO3	+	
valinillin	C8H8O3		+
4-methyl-3-oxoadipate	C7H10O5		+
4-methyl-3-oxoadipate-enol-lactone	C7H8O4		+
D-5-benzyl-hydantoin	C10H10N2O2		+
5-hydroxyindole thiazolidine carboxylate	C13H14N2O3S		+
5-hydroxyindole acetate	C10H9NO3		+
5- hydroxyindole acetaldehyde	C10H9NO2		+
34-dihydroxypenylacetaldehyde	C8H8O3		+
Indole pyruvate	C11H9NO3		+
3-indolylglycolaldehyde	C10H9NO2		+
enol-phenylpyruvate	C9H8O3		+
sinapate	C11H12O5		+
5-hydroxy-ferulic acid	C10H10O5		+
N-acetylmuramate	C11H19NO8		+
N-acetyl-β-D-galactosamine	C8H15NO6		+
scopoletin	C10H8O4		+
6-hydroxy-2-cyclohexen-one-carboxylate	C7H8O4		+
coumarinate	C9H8O3		+
trans-caffeate	C9H8O4		+
vanillyl mandelate	C9H10O5		+
3-methoxy-4-hydroxyphenylglycol	C9H12O4		+
2-hydroxychromene-2-carboxylate	C10H8O4		+
7,8-dihydrolumazine	C6H6N4O2		+
benzyl acetate	C9H10O2		+

Table S7: Growth rate and yield of *Leptocylindrus danicus* and *Thalassiosira pseudonana* amended with 4-amino-5-hydroxymethyl-2-methylpyrimidine (HMP), 4-methyl-5-(β-hydroxyethyl)thiazole (HET), HMP+HET and vitamin B1 and a control. Give are mean values (N=3) ± standard deviation and significant differences relative to the control (Student's t-test). * $p < 0.05$; ** $p < 0.01$; *** $p < 0.001$

Alga	parameter	HET	HMP	HET & HMP	Vitamin B1	Control
L. danicus	Growth (μ)	0.183 ± 0.002 **	0.188 ± 0.006 ***	0.168 ± 0.008	0.158 ± 0.008	0.157 ± 0.004
	Growth yield	1506 ± 54 ***	1429 ± 29 ***	1232 ± 152	1062 ± 29	1114 ± 44
T. pseudonana	Growth (μ)	0.397 ± 0.011 ***	0.387 ± 0.008 ***	0.368 ± 0.002 ***	0.363 ± 0.007 **	0.325 ± 0.006
	Growth yield	503 ± 8	499 ± 19	518 ± 9	507 ± 33	509 ± 17

Supplementary Figures

Supplementary Figure S1

Cell numbers and standard deviation (N=3) of *D. shibae* (upper panel) and *P. inhibens* (lower panel) grown on glutamate, glucose and acetate or succinate, respectively, until the stationary phase was reached. Incubation times of the three carbon sources were different.

Supplementary Figure S1:

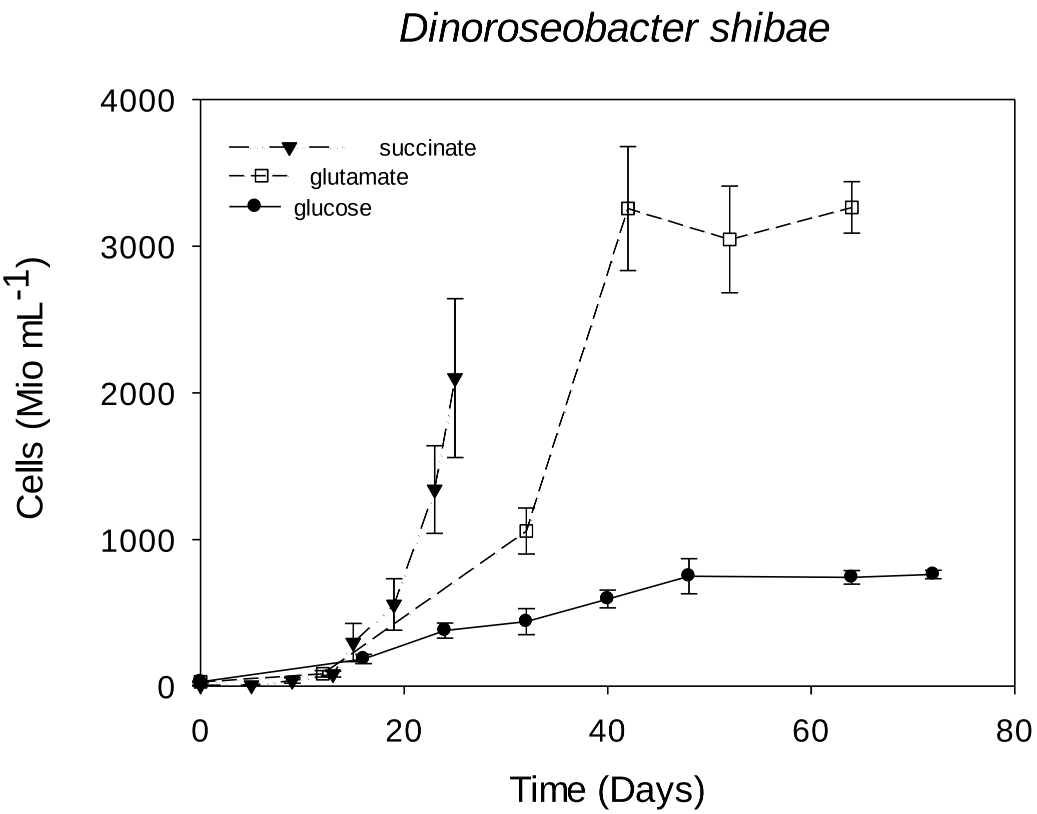

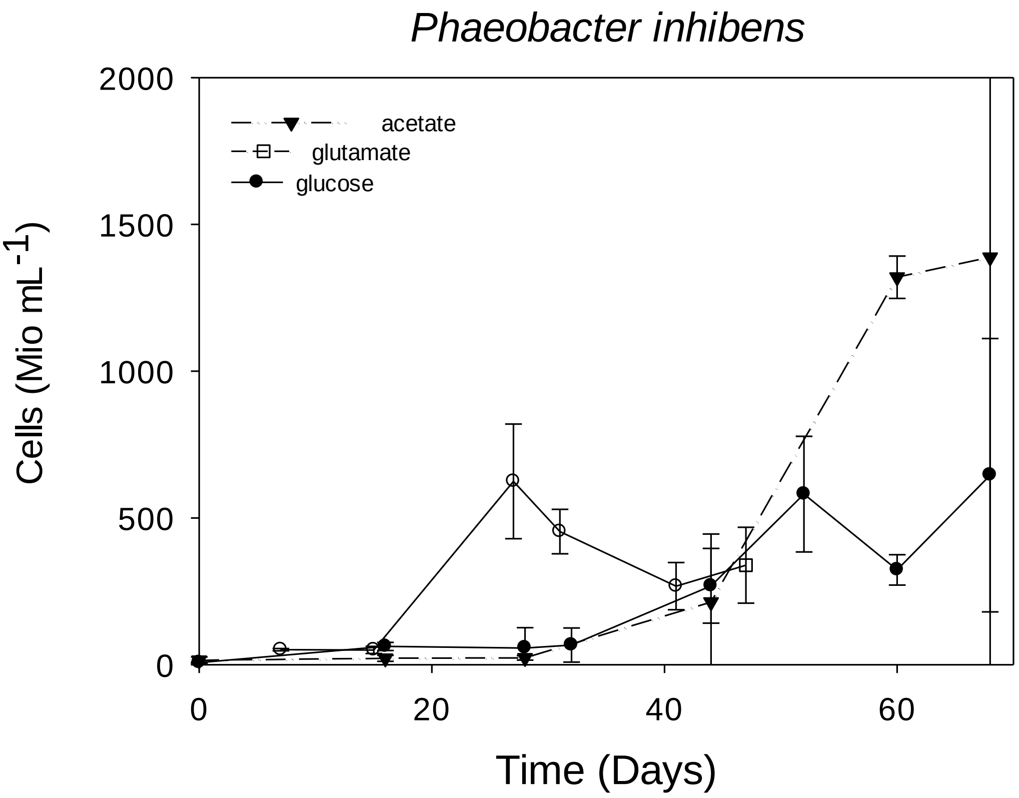

Supplement: Supplementary file 1 [file Presentation1.PDF]
